# Diagnosis and treatment of giant lateral abdominal wall haematoma after blunt trauma: a case report

**DOI:** 10.1186/1757-1626-2-9358

**Published:** 2009-12-19

**Authors:** Sumanta Dutta, Pandanaboyana Sanjay, Mike L Jones

**Affiliations:** 1 University Department of Surgery, University of Glasgow-Faculty of Medicine, Royal Infirmary, Glasgow, UK; 2 Department of Surgery & Molecular Oncology, Ninewells Hospital & Medical School, University of Dundee, Dundee, UK

## Abstract

Lateral abdominal wall haematoma after blunt trauma that require surgery is rare. They usually present with pain, bruising and swelling after trauma.

We report a case of a fit and healthy young girl who developed a large lateral abdominal wall haematoma following blunt trauma. Initially the haematoma was managed conservatively, however in view of increasing size surgical removal was undertaken. Post operatively the patient developed a small seroma and which was subsequently drained under ultrasound guidance. A thorough review of the literature has identified there are various options of treatment for patients with lateral abdominal wall haematoma. We conclude that management of giant traumatic lateral abdominal wall haematoma can be challenging, some will eventually need surgical intervention.

## Background

Although the majority of haematoma of the lateral abdominal wall after blunt trauma are managed conservatively, some may need surgical management. We present a case of post-traumatic lateral abdominal wall haematoma that increased in size over few months after the initial injury and eventually required surgical excision.

## Case presentation

A 21-year-old healthy young girl suffered a ski-ing injury to her right flank. She is a non-smoker and does not have any significant medical or family history. An ultrasound scan performed three months after the accident showed a large subcutaneous haematoma in right posterior paraspinal region. There was no significant Doppler flow in that area. She was initially managed conservatively. The haematoma appeared to increase in size and she subsequently had a CT scan, which showed a 13 × 10 × 20 cm well defined low density cystic mass occupying the right flank and descending posteriorly to involve the gluteal muscles and upper thigh. The swelling had fine septations but was otherwise uniform in echo density and there was no infiltration into the deep muscular layers. The features were consistent with a resolving haematoma with no evidence of acute haemorrhage. Her routine haematological investigations including haemoglobin on admission were within normal limits. She was investigated for bleeding diathesis and there was no evidence of one. There was no family history of bleeding diathesis.

**Figure 1:**
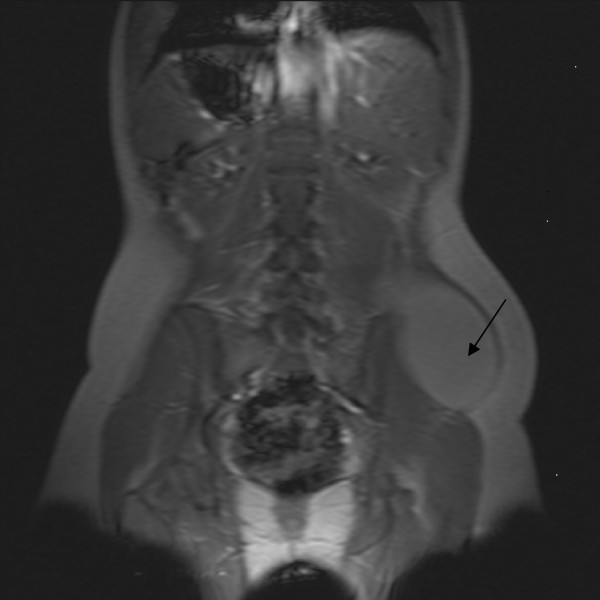
**MRI scan in the coronal plane showing the lateral wall haematoma**.

**Figure 2:**
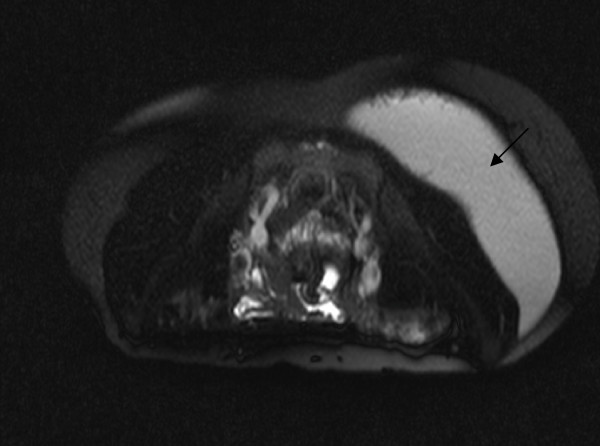
**MRI in sagital plane showing the collection was superficial to the muscles of the back and the gluteal region but deep to the posterior layer of the thoraco-lumbar fascia**.

An attempted aspiration under local anaesthesia was unsuccessful. In view of the increasing swelling and discomfort an MRI scan of the abdomen and pelvis (Figure 1 and 2) was obtained. This revealed a large well-defined ovoid fluid collection in the left side of the back extending inferiorly into the left gluteal region. It measured 14 × 10 cm in transverse dimension and 20 cm in cranio-caudal extent. The collection appeared to be situated superficial to the muscles of the back and the gluteal region but deep to the posterior layer of the thoraco-lumbar fascia. Multiple irregular septations were noted.

**Figure 3:**
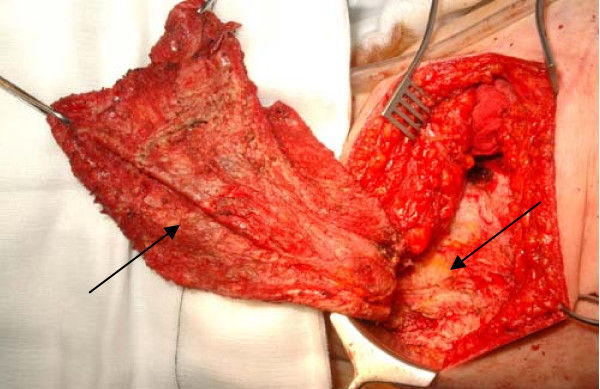
**Per operative picture showing the wall of the haematoma and the cavity, which is superficial to the muscle layer**.

All features were in keeping with a large sub-fascial organised haematoma. The source of bleeding was probably disrupted sub-fascial veins from the force of blunt trauma. In view of the ongoing pain and disfigurement the haematoma plus organised capsule was evacuated and excised enbloc. 1.5 litres of old blood was evacuated from the organised haematoma (Figure 3). Two suction drains were left in situ for two weeks. The patient developed a small seroma 7 days after removing the drains. This was resolved by a placement of a further suction drain under ultrasound guidance for a further 14 days.

## Discussion

Haematomas of the abdominal wall can be divided in the common rectus sheath haematomas and the less frequent lateral abdominal wall haematomas. Rectus sheath haematoma can develop spontaneously in patients with severe hypo-coagulability such as haemophilia, von-willebrand disease or sometime anticoagulant therapy. It is also a rare complication of pregnancy, laparoscopic cholecystectomy or even abdominal insulin injections [1]. Spontaneous rectus sheath haematoma caused by rupture of inferior epigastric artery has also been reported [2].

Lateral wall haematomas are uncommon and there are few reports or studies. They are classified into two groups- slow growing (oozing from muscle surface/venous bleed) and rapidly growing (arterial bleed usually from deep circumflex iliac artery) haematoma. Lefere et al [3] reported an expanding haematoma of the lateral abdominal wall after blunt trauma and Ilkgul et al [4] reported a case of post-traumatic lateral abdominal wall haematoma with non specific findings. There has been reported cases of iatrogenic haematoma from deep circumflex iliac artery as a result of trocar placement during laparoscopic surgery [5] as well as spontaneous haemorrhage from the same artery by Katsumori et al [6] and Shimizu et al[7].

Diagnosis can be made clinically by obvious swelling/bruising or may be non-specific findings like chronic anaemia, fever and plural effusion [4]. Imaging studies like ultrasound/CT/MRI helps to confirm the diagnosis by differentiating between rectus sheath and lateral abdominal wall compartment haematomas as well as help to exclude the intra abdominal haemorrhage.

The majority of haematomas can be treated conservatively. CT and MRI provide accurate information with regards to characteristics of the haematoma. There may be role for angiography (with or without embolisation) in patients with an acutely expanding haematoma with arterial bleed causing haemodynamic instability [3,6,7]. Slow growing lateral wall haematoma may be less obvious initially mainly because of their anatomical location and can be managed conservatively in most of the cases. But progressive disfigurement and discomfort necessitated surgical intervention in our case. Postoperative seroma is not uncommon and there are different modalities of treatments available with varying degree of success. A recent study showed sclerotherapy as a promising treatment in post mastectomy seromas [8]. In our case it was managed by radiology-guided drainage.

## Conclusion

The majority of haematomas resulting from blunt trauma in our experience are managed conservatively. In the acute setting, our indications for an intervention, either radiological or surgical would be an acutely expanding haematoma with suspicion of underlying major vessel injury associated with haemodynamic instability. Our experience with this case suggests initial conservative management is feasible if the patients are haemodynamically stable. However, during follow up a low threshold for surgical intervention is recommended for un-resolving haematomas. A combination of investigation (CT/MRI) and interventions (surgical/radiological) are required to ensure complete resolution of these haematomas.

## Consent

Written informed consent was obtained from the patient for publication of this case report and any accompanying images. A copy of the written consent is available for review by the Editor-in-Chief of this journal.

## Competing interests

The authors declare that they have no competing interests.

## Authors' contributions

SD collected the data including images, performed literature search and drafted the manuscript. PS reviewed the literature and revised the draft critically. MLJ revised and approved the final version of the manuscript. All authors read and approved the manuscript.

## References

[B1] Monsein LH Davis M Radionuclide imaging of a rectus sheath hematoma caused by insulin injections Clin Nucl Med 1990 15 8 539 541 10.1097/00003072-199008000-00002 2143969

[B2] Zainea GG Jordan F Rectus sheath hematomas: their pathogenesis, diagnosis, and management Am Surg 1988 54 10 630 633 2972238

[B3] Lefere P Gryspeerdt S Van Holsbeeck B Baekelandt M Diagnosis and treatment of expanding haematoma of the lateral abdominal wall after blunt abdominal trauma Eur Radiol 1999 9 8 1553 1555 10.1007/s003300050883 10525864

[B4] Ilkgul O Ozden S Ozsoy Y Yoleri L Erhan Y Aydede H Late diagnosis of a lateral abdominal wall hematoma presenting with nonspecific findings: report of a case Ulus Travma Acil Cerrahi Derg 2007 13 4 316 318 17978915

[B5] Reid GD Cooper MJ Parker J Implications for port placement of deep circumflex iliac artery damage at laparoscopy J Am Assoc Gynecol Laparosc 1999 6 2 221 223 10.1016/S1074-3804(99)80108-X 10226138

[B6] Katsumori T Nakajima K A case of spontaneous hemorrhage of the abdominal wall caused by rupture of a deep iliac circumflex artery treated by transcatheter arterial embolization Eur Radiol 1998 8 4 550 552 10.1007/s003300050432 9569320

[B7] Shimizu T Hanasawa K Yoshioka T Mori T Kajinami T Yokoyama K Sho K Tani T Spontaneous hematoma of the lateral abdominal wall caused by a rupture of a deep circumflex iliac artery: report of two cases Surg Today 2003 33 6 475 478 10.1007/s10595-002-2512-1 12768378

[B8] Throckmorton AD Askegard-Giesmann J Hoskin TL Bjarnason H Donohue JH Boughey JC Degnim AC Sclerotherapy for the treatment of postmastectomy seroma Am J Surg 2008 196 4 541 544 10.1016/j.amjsurg.2008.06.020 18809059

